# Glucocorticoid-Induced Reversal of Interleukin-1β-Stimulated Inflammatory Gene Expression in Human Oviductal Cells

**DOI:** 10.1371/journal.pone.0097997

**Published:** 2014-05-21

**Authors:** Stéphanie Backman, Alexandra Kollara, Robin Haw, Lincoln Stein, Theodore J. Brown

**Affiliations:** 1 Department of Obstetrics and Gynecology, University of Toronto, Toronto, Ontario, Canada; 2 Lunenfeld-Tanenbaum Research Institute, Mt. Sinai Hospital, Toronto, Ontario, Canada; 3 Ontario Institute for Cancer Research, Informatics and Bio-Computing, Toronto, Ontario, Canada; Baylor College of Medicine, United States of America

## Abstract

Studies indicate that high-grade serous ovarian carcinoma (HGSOC), the most common epithelial ovarian carcinoma histotype, originates from the fallopian tube epithelium (FTE). Risk factors for this cancer include reproductive parameters associated with lifetime ovulatory events. Ovulation is an acute inflammatory process during which the FTE is exposed to follicular fluid containing both pro- and anti-inflammatory molecules, such as interleukin-1 (IL1), tumor necrosis factor (TNF), and cortisol. Repeated exposure to inflammatory cytokines may contribute to transforming events in the FTE, with glucocorticoids exerting a protective effect. The global response of FTE cells to inflammatory cytokines or glucocorticoids has not been investigated. To examine the response of FTE cells and the ability of glucocorticoids to oppose this response, an immortalized human FTE cell line, OE-E6/E7, was treated with IL1β, dexamethasone (DEX), IL1β and DEX, or vehicle and genome-wide gene expression profiling was performed. IL1β altered the expression of 47 genes of which 17 were reversed by DEX. DEX treatment alone altered the expression of 590 genes, whereas combined DEX and IL1β treatment altered the expression of 784 genes. Network and pathway enrichment analysis indicated that many genes altered by DEX are involved in cytokine, chemokine, and cell cycle signaling, including NFκΒ target genes and interacting proteins. Quantitative real time RT-PCR studies validated the gene array data for *IL8*, *IL23A*, *PI3* and *TACC2* in OE-E6/E7 cells. Consistent with the array data, Western blot analysis showed increased levels of PTGS2 protein induced by IL1β that was blocked by DEX. A parallel experiment using primary cultured human FTE cells indicated similar effects on *PTGS2*, *IL8*, *IL23A*, *PI3* and *TACC2* transcripts. These findings support the hypothesis that pro-inflammatory signaling is induced in FTE cells by inflammatory mediators and raises the possibility that dysregulation of glucocorticoid signaling could contribute to increased risk for HGSOC.

## Introduction

High-grade serous ovarian cancer (HGSOC) is the most common of the epithelial ovarian cancer histotypes and almost invariably presents as late stage disease associated with poor prognosis [1]. While traditionally thought to derive from the ovarian surface epithelium, recent studies indicate that HGSOCs likely originate in the fallopian tube epithelium (FTE) [2], [3]. Women with germline mutations in breast cancer susceptibility genes 1 or 2 (*BRCA1* or *2*) are at high risk of developing HGSOC and often elect to undergo bilateral salpingoophorectomy to reduce this risk [4]. Histomorphological examination of fallopian tubes from these women has revealed putative HGSOC precursor lesions [3], [5]. These include regions of FTE exhibiting strong p53 immunostaining, reflective of *TP53* mutations characteristic of HGSOCs, and tubal intraepithelial carcinomas (TICs), which are occult *in situ* adenocarcinomas [6], [7], [8]. TICs have been found in more than half of patients presenting with HGSOC [7], [9], [10], [11] and share identical *TP53* mutations with the invasive tumor, supporting the concept that they are clonally related [8].

Risk factors for epithelial ovarian cancer are associated with increased lifetime ovulatory years [12], [13], [14], [15], which have led to the concept that ovulation may contribute to malignant transformation of adnexal epithelia. Ovulation is a localized acute inflammatory event during which fimbrial epithelial cells are exposed to follicular fluid containing a complex combination of inflammatory molecules [16]. Prolonged exposure to pro-inflammatory signaling can result in DNA adduct formation, increasing the incidence of gene mutations that can lead to malignant transformation [17], [18], [19]. Glucocorticoids have been shown to exert anti-inflammatory effects in several tissues [20]. We have previously shown that *BRCA1* enhances glucocorticoid receptor signaling and found evidence of suppressed glucocorticoid activity in luteal phase FTE from *BRCA1* mutation carriers relative to control patients [21]. However, anti-inflammatory activity of glucocorticoids does not occur in all cell types. For example, a stimulatory rather than inhibitory effect of glucocorticoids on expression of prostaglandin-endoperoxide synthase 2 (PTGS2), the rate-limiting enzyme in prostaglandin production, has been shown in amnion fibroblasts [22], placental cytotrophoblasts [23], cardiomyocytes [24], and nasal polyps [25].

Human FTE cells have been shown to respond to IL1 with increased IL-8 expression [26], the global response of fallopian epithelial cells to inflammatory cytokines and/or glucocorticoids has not been investigated. In this study, we used an immortalized human oviductal cell line (OE-E6/E7) to assess changes in gene expression induced by Interleukin-1β (IL1β), a pro-inflammatory cytokine, implicated in ovulation [27], and dexamethasone (DEX), a glucocorticoid receptor agonist, to determine whether glucocorticoid signaling alters the response to IL1β in these cells. OE-E6/E7 cells were derived from ampullary tubal epithelial cells of a patient who underwent surgery for uterine fibromyoma and were immortalized using HPV16 E6/E7 [28]. These cells have been extensively characterized and shown to exhibit properties consistent with oviductal secretory epithelial cells [28], [29], [30]. Thus, these cells serve as a useful model to explore mechanisms that could relate to early carcinogenesis.

## Materials and Methods

### Cell Culture

OE-E6/E7 cells [28] were obtained from Dr. William S.B. Yeung, University of Hong Kong (Hong Kong, China). BT20 human breast cancer cells were obtained from ATCC (Manassas, VA). Both cell lines were maintained in Dulbecco's Modified Eagle's medium supplemented with 10% fetal bovine serum (FBS), 100 U/ml penicillin and 100 µg/ml streptomycin (all from Invitrogen, Burlington, ON). A human fallopian tube sample was obtained from a patient undergoing prophylactic bilateral salpingoophorectomy for a *BRCA2* mutation at Mount Sinai Hospital (MSH). The tissue collection and use was made with patient informed consent and was approved by the MSH Research Ethics Board. Epithelial cells were derived from the tissue and grown in primary culture on collagen-coated transwell membranes as previously described by Fotheringham at al [31]. All cells were grown in a humidified incubator at 37°C and 5% CO_2_. DEX (Sigma, St. Louis, MO) was dissolved in ethanol at 1 mM. Recombinant human IL1β and TNFα (R&D Systems, Minneapolis, MN) were reconstituted in phosphate-buffered saline containing 0.1% bovine serum albumin to 25 µg/ml and 100 µg/ml, respectively. Stock solutions were diluted with culture medium just prior to use to achieve final stated concentrations. Cells were transferred to medium containing 0.5% charcoal-stripped FBS 18 h before initiating hormone or cytokine treatments.

### Western Blot Analysis

Cells were collected in RIPA lysis buffer (150 mM NaCl, 50 mM HEPES pH 7.25, 1% Triton X-100, 0.1% SDS, 1% sodium deoxycholate) containing Complete protease inhibitor cocktail (Roche Diagnostics, Laval, QC). Clarified lysates were collected by centrifugation at 20,000 *g* for 15 min at 4°C and total protein was quantified using a BCA protein assay (Pierce, Rockford, IL) following the manufacturer's protocol. Aliquots of lysates containing 15 to 20 µg of total protein were subjected to Western blot analysis as described previously [32] using anti-PTGS2 mouse monoclonal antibody (1∶1000; Cayman Chemicals, Ann Arbor, MI) or anti-glucocorticoid receptor rabbit polyclonal (1∶500; Abcam, Cambridge, MA) or anti-tubulin mouse monoclonal antibody (1∶5000; Sigma). Immunoreactive band intensities were quantified using Molecular Dynamics Image Quant version 5.0 software. Statistical analysis was performed using one-way ANOVA followed by Student-Newman-Keuls post-hoc multiple comparison test (SigmaStat, Systat Software Inc, Chicago, IL). Comparisons were considered statistically significant at p<0.05.

### Gene Expression Analysis

Total RNA extracted using Trizol reagent (Invitrogen) and verified for integrity and purity, was labeled using Illumina TotalPrep RNA amplification kit (Ambion, Austin, TX) as per manufacturer's instructions. The cRNA generated was hybridized to Illumina HT-12 v4.0 BeadChips at 58°C for 18 h. The BeadChips were then washed, stained, and scanned using iScan (Illumina). Data files were quantified in GenomeStudio v2011.1 (Illumina), quantile normalized using the lumi package of Bioconductor [33], and filtered using varFilter to retain the 10% most variable probes. Data for multiple probes targeting the same gene were collapsed to their median value and annotated with gene names using GenePattern [34]. The collapsed data were analyzed using the linear models for microarray analysis (LIMMA) package [35] to identify differentially expressed genes. To understand the relationship among differentially expressed genes further, we performed a network-based analysis using the Reactome Functional Interaction (FI) Network plugin for Cytoscape [36]. The gene expression profiling data can be accessed from the NCBI Gene Expression Omnibus (GEO; http://www.ncbi.nlm.nih.gov/geo. Accessed 2014 May 1), accession number GSE54608.

### Quantitative real-time RT-PCR (RT-qPCR)

Total RNA from OE-E6/E7 and primary FTE cells was isolated using Trizol reagent (Invitrogen) following the manufacturer's protocol. Traces of genomic DNA from RNA isolated from OE-E6/E7 cells were removed using a TURBO DNA-free Kit (Applied Biosytems, Foster City, CA) following the manufacturer's protocol. RNA from primary FTE cells was purified using the RNeasy MinElute Clean up kit (Qiagen, Toronto, ON). RNA purity and integrity was determined using a bioanalyzer (Agilent Technologies, Inc, Mississauga, ON) and only samples with a RNA integrity number greater than 9 were used. The RNA was then reverse transcribed using Superscript III Reverse Transcriptase and random hexamers (Invitrogen) following the manufacturer's protocol. Primers were designed to span an intron/exon boundary using Primer3 (v.0.4.0.) and NCBI Primer-BLAST programs (Table 1) and their specificity was verified by BLAST analysis. Real-time qPCR was performed using a Perkin Elmer-JAMUS automated liquid handling system (Perkin Elmer, Waltham, MA) and a C1000 Thermal cycler (Bio-Rad, Hercules, CA) with LuminoCt SYBER Green (Sigma) detection using a total reaction volume of 5 µl containing 2.5 µl SYBER Green, 300 nM of each primer pair and 75 ng cDNA in 384 well plate. The cycling conditions were 95°C for 30 sec, 95°C for 5 sec, and 60°C for 20 sec. At the end of the PCR cycle, a dissociation curve was performed to confirm amplification of a single product. Each template was run with reference genes (Glyceraldehyde-3-phosphate dehydrogenase, *GAPD*; Beta-2-microglobulin, *B2M*; and Tyrosine 3-monooxygenase/tryptophan 5-monooxygenase activation protein, Zeta polypeptide, *YWHAZ*; and/or TATA-binding protein, *TBP*). RT-qPCR data were analyzed using Bio-Rad CFX manager 2.0 software (Bio-Rad) and the targeted Cq values were normalized to *TBP* (OE-E6/E7 cells) or the geometric mean of *TBP, GAPD, B2M* and *YWHAZ* values using standard curves for each gene to account for any possible differences in primer efficiency. Statistical analysis was performed using two-way ANOVA followed by Student-Newman-Keuls post-hoc multiple comparison test. In cases of inhomogeneity of variance, data were log-transformed or analyzed using Kruskal-Wallis ANOVA on ranks followed by Student-Newman-Keuls post-hoc multiple comparison test. Comparisons were considered statistically significant at p<0.05.

**Table 1:** List of primers used for RT-qPCR.

Gene ID	Primer sequence	NCBI accession #
*PTGS2*	F: GTTCCCACCCATGTCAAAAC	NM_000963
	R: ATTCCGGTGTTGAGCAGTT	
*IL23A*	F: CAAGGACTCAGGGACAACAG	NM_016584
	R: GCTCCCCTGTGAAAATATCC	
*IL8*	F: CTCTCTTGGCAGCCTTCCT	NM_000584
	R: GGGTGGAAAGGTTTGGAGTA	
*PI3*	F: ATCGTGGTGGTGTTCCTCAT	NM_002638
	R: ACGGGATCTTGTCCATTGAA	
*TACC2*	F: TCAGGAGAGCCCTGTCAAGT	NM_206861
	R: GTTTTTCGCAGCAGTGTTCA	
*TBP*	F: TGCACAGGAGCCAAGAGTGAA	NM_003194
	R: CACATCACAGCTCCCCACCA	
*GAPD*	F: CGAGCCACATCGCTCAGA	NM_002046
	R: AGTTAAAAGCAGCCCTGGTGA	
*B2M*	F: CTCCGTGGCCTTAGCTGTG	NM_004048
	R: TTGGAGTACGCTGGATAGCCT	
*YWHAZ*	F: ACGTCCCTCAAACCTTGCTT	NM_003406
	R: GGCCTTCTGAACCAGCTCAT	

F =  forward, R =  reverse.

## Results

**Figure 1:**
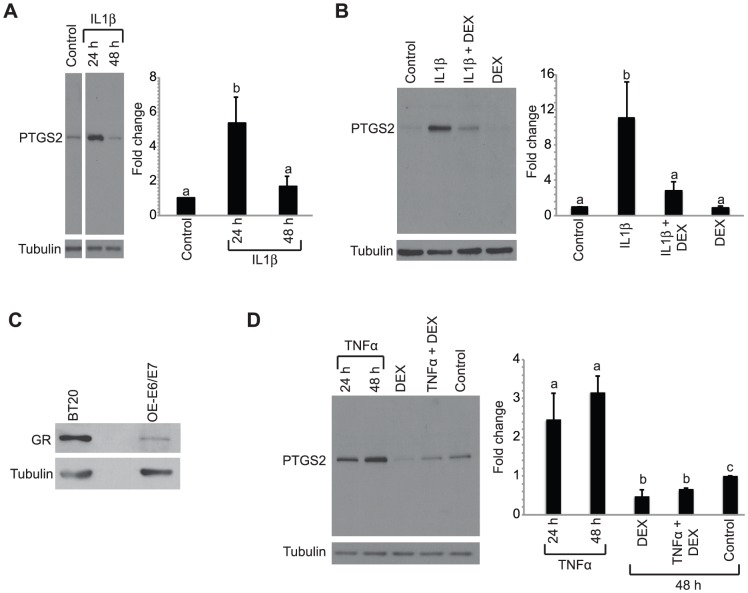
Increased PTGS2 levels in OE-E6/E7 cells by IL1β and TNFα treatment is blocked by DEX. A, Cells were treated with 50/ml IL1β for 24 h or 48 h and Western blot analysis was performed for PTSG2 and tubulin. B, Cells were treated with 10 nM DEX or vehicle 30 h prior to treatment with IL1β or vehicle and harvested 24 h later. Western blot analysis was performed for PTGS2 and tubulin. C, Western blot analysis was performed on OE-E6/E7 cells for glucocorticoid receptor and tubulin levels with BT20 breast cancer cells used as positive control. D, Cells were treated with DEX or vehicle 30 h prior to treatment with TNFα or vehicle. Cells treated with TNFα alone were harvested at 24 and 48 h after treatment and cells treated with DEX+TNFα were harvested at the 48 h time point. Western blot analysis was performed for PTGS2 and tubulin. Histograms summarize quantification of PTGS2 levels normalized to tubulin in 3 to 6 immunoblots. Bars represent the mean ± SEM relative to control. Bars with different letters are statistically different from one another as determined by ANOVA followed by a Student-Newman-Keuls post-hoc multiple comparison test (*p*<0.05).

We first established that OE-E6/E7 cells respond to IL1β treatment with increased PTGS2 expression. Western blot analysis showed a more than 5-fold increase in levels of PTGS2 at 24 h, but not 48 h after treatment with 50 ng/ml IL1β, relative to control-treated cells (Figure 1A). Pre-treatment of cells with 10 nM DEX reversed the IL1β-induced increase at 24 h but did not affect uninduced PTGS2 levels (Figure 1B). Western blot analysis indicated OE-E6/E7 cells express glucocorticoid receptor but at levels below that of BT20 used as a positive control [37] (Figure 1C). A similar inhibitory effect on PTGS2 was observed with TNFα (Figure 1D). Treatment of cells with 100 ng/ml TNFα increased PTGS2 levels with the highest levels observed at 48 h after treatment. TNFα induction of PTGS2 at 48 h was blocked by pre-treatment with DEX (Figure 1D).

**Figure 2:**
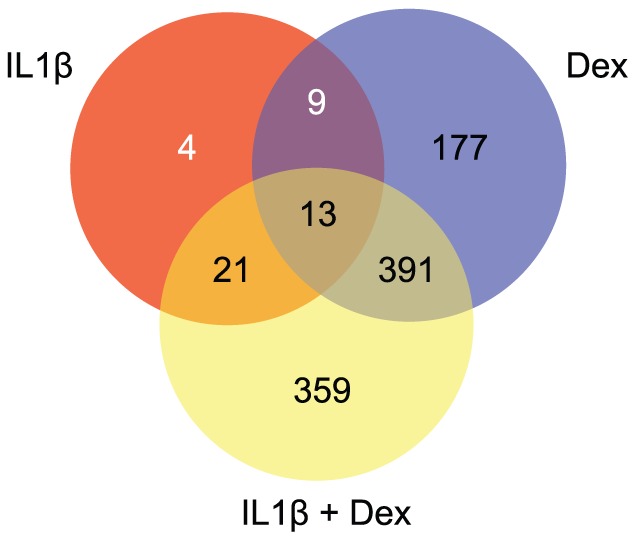
Number and overlap of genes differentially expressed due to IL1β, DEX, and IL1β+DEX treatment. Cells were treated with 10/ml IL1β or vehicle and harvested 18 h later. Total RNA was extracted and gene expression profiling was performed on Illumina HT-12 v4.0 BeadChips. The venn diagram shows the overlap of genes differentially expressed by the different treatments relative to control treated cells.

The global impact of IL1β and glucocorticoid treatment on gene expression in OE-E6/E7 cells was determined by whole genome microarray analysis. Cells were treated with DEX or vehicle 30 h prior to treatment with IL1β or vehicle. Cells were harvested 18 h after IL1β treatment and RNA was extracted, labeled, and hybridized to Illumina HT-12 v4.0 BeadChip arrays. Genes differentially expressed between treatment groups and the control group were identified by t-test with correction for multiple testing. Altogether, a statistically significant alteration due to treatment was detected for expression of 974 of the 26,737 genes. Relative to control, IL1β treatment altered the expression of 47 genes, whereas DEX altered the expression of 590 genes (Table S1). Combined IL1β and DEX treatment altered the expression of 784 genes as compared to control-treated cells (Table S1). Of the 47 genes with altered expression due to IL1β treatment, 4 were unique to IL1β and 9 were also altered by DEX alone (Figure 2). IL1β increased the expression of 7 and decreased the expression of 2 of these 9 genes with DEX treatment alone having an opposite effect on their expression relative to control-treated cells (Table 2). None of the 13 genes were altered by combined IL1β and DEX treatment, as compared to control group (Figure 2). Thus, DEX treatment opposed the effect of IL1β on the expression of these 13 genes. Thirteen genes were commonly regulated by all three treatment groups relative to the control group (Figure 2). Pre-treatment with DEX reversed the impact of IL1β on 4 while enhancing the effect on 8 of these 13 genes (Table 2).

**Table 2:** Genes with altered expression due to IL1β treatment.

		Log2 Fold-Change*		
Gene ID	Gene Name	IL1β	DEX	IL1β+DEX
	**13 genes altered in all 3 treatment groups**			
*NGLY1*	N-glycanase 1	0.35	0.30	0.43
*TRIML2*	Tripartite motif family-like 2	0.63	−1.03	−0.67
*FOXQ1*	Forkhead box Q1	0.97	0.69	1.58
*CCL20*	Chemokine (C-C motif) ligand 20	0.43	0.49	0.84
*C7ORF10*	Chromosome 7 open reading frame 10	0.40	1.11	0.98
*EBI3*	Epstein-Barr virus induced 3	0.46	0.79	1.53
*GJB2*	Gap junction protein, beta 2	0.75	−0.39	0.36
*SYTL2*	Synaptotagmin-like 2	−0.85	−1.22	−1.58
*PLD5*	Phospholipase D family, member 5	−0.73	2.59	2.25
*PRSS23*	Protease, serine, 23	−0.64	−0.56	−1.53
*MAMDC2*	MAM domain containing 2	−0.55	−0.59	−1.38
*LTB*	Lymphotoxin beta	−0.81	−0.88	−1.92
*CAMK2N1*	Ca^++^/calmodulin-dependent protein kinase II inhibitor 1	−0.59	0.91	0.38
	**21 genes altered by IL1β and by IL1β+Dex**			
*BATF3*	Basic leucine zipper transcription factor, ATF-like 3	0.64	NS	0.91
*SAT1*	Spermidine/spermine N1-acetyltransferase 1	0.45	NS	0.29
*CES1*	Carboxylesterase 1	0.81	NS	0.88
*E2F2*	E2F transcription factor 2	0.71	NS	0.45
*ST6GAL1*	ST6 beta-galactosamide alpha-2,6-sialyltransferase 1	0.47	NS	0.45
*CXCL5*	Chemokine (C-X-C motif) ligand 5	0.70	NS	0.71
*APPL2*	Adaptor protein, phosphotyrosine interaction, PH domain and leucine zipper containing 2	0.37	NS	0.24
*CXCL6*	Chemokine (C-X-C motif) ligand 6	0.65	NS	0.60
*IL17C*	Interleukin 17C	1.22	NS	1.45
*ARG2*	Arginase, type II	0.79	NS	0.68
*PI3*	Peptidase inhibitor 3	1.18	NS	1.66
*RAB38*	RAB 38, member RAS oncogene family	0.68	NS	0.68
*FLJ10986*	FGGY, Carbohydrate kinase domain containing	0.64	NS	0.40
*FAM19A3*	Chemokine (C-C motif)-like protein TAFA-3	−0.68	NS	−0.84
*TMEM166*	Transmembrane protein 166	−0.61	NS	−0.40
*OXTR*	Oxytocin receptor	−0.71	NS	−0.67
*SPOCK2*	Testican	−0.41	NS	−0.58
*CXCL1*	Chemokine (C-X3-C motif) ligand 1	−0.99	NS	−0.69
*ITGA3*	Integrin, alpha 3	−0.50	NS	−0.64
*C15ORF52*	Chromosome 15 open reading frame 52	−0.34	NS	−0.26
*GBP4*	Guanylate binding protein 4	−0.62	NS	−0.45
	**9 genes altered by IL1β and by DEX**			
*PTGS2*	Prostaglandin-endoperoxide synthase 2	1.10	−1.57	NS
*NFKBIZ*	I-Kappa-B-zeta	0.46	−0.32	NS
*FGFRL1*	Fibroblast growth factor receptor-like 1	0.43	−0.31	NS
*S100A9*	S100 calcium binding protein A9	1.19	−0.96	NS
*LOC728454*	Similar to Beta-defensin 2 precursor	0.69	−0.83	NS
*PTPN20*	Protein tyrosine phosphatase, non-receptor type 20	0.51	−0.44	NS
*DEFB4*	Defensin, beta 4	0.69	−0.76	NS
*LI10RB*	Interleukin 10 receptor, beta	−0.50	0.42	NS
*NFKBIE*	I-Kappa-B-epsilon	−0.47	0.45	NS
	**4 genes altered by IL1β only**			
*LXN*	Latexin	0.63	NS	NS
*MAGED1*	Melanoma antigen family D, 1	0.40	NS	NS
*CCDC24*	Coiled-coil domain containing 24	−0.43	NS	NS
*IL1R2*	Interleukin 1 receptor, type II	−0.52	NS	NS

*Values represent log2 fold change relative to control-treated cells with adjusted p-value <0.05.

NS =  non significant (adjusted p-value >0.05).

Twenty-one genes were common between the IL1β+DEX and IL1β alone versus control comparisons, but not DEX alone versus control (Figure 2 and Table 2). The impact of treatment on the expression of these genes reflects IL1β since DEX neither further enhanced nor inhibited the IL1β effect. This was confirmed by direct comparison of the IL1β alone and IL1β+DEX treatment groups (data not shown).

**Figure 3:**
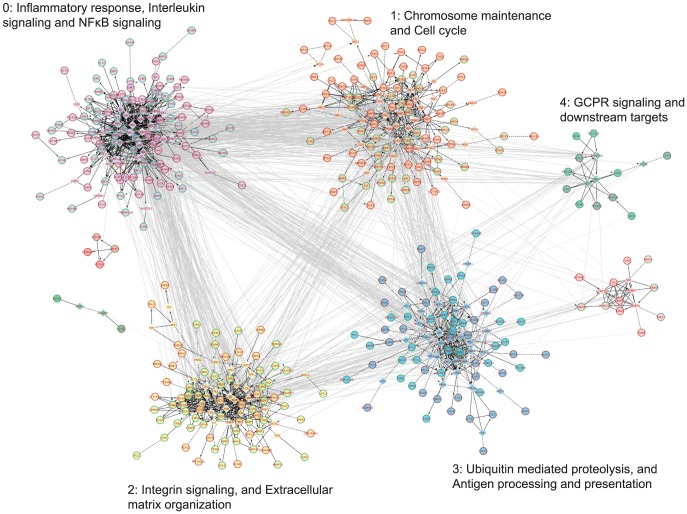
Network analysis of differentially expressed genes in human FTE OE-E6/E7 cells treated with DEX. Module annotations were performed with false discovery rate (FDR)<0.05. Genes up-regulated in human FTE OE-E6/E7 cells treated with DEX are depicted by red outer circles, whereas genes down-regulated in human FTE OE-E6/E7 cells treated with DEX are depicted by green outer circles. Linker genes are depicted as diamond shaped nodes. Direct activating or inhibitory interactions are indicated with the symbols → and -|, respectively. Indirect interactions involving additional proteins are depicted with dashed lines.

**Figure 4:**
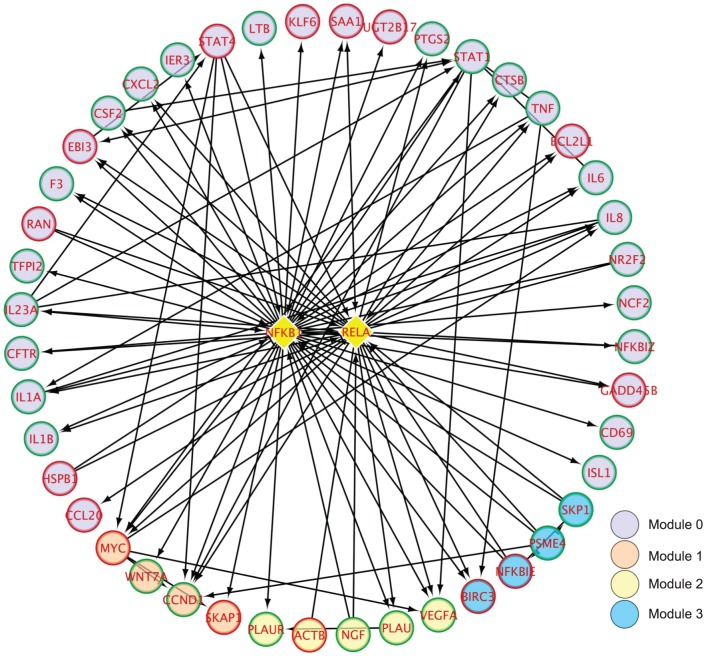
DEX affected NFκB target genes and interacting proteins across four network modules identified by network-based analysis. Genes up-regulated by DEX are depicted by red outer circles, whereas genes down-regulated by DEX are depicted by green outer circles. NFκB subunits are depicted by yellow diamonds. Direct activating or inhibitory interactions are indicated with the symbols → and -|, respectively. Indirect interactions involving additional proteins are depicted with dashed lines.

DEX treatment altered the expression of a greater number of genes than IL1β. We used the Reactome Functional Interaction (FI) Network to explore the relationships among differentially expressed genes in OE-E6/E7 cells treated with DEX [36]. In brief, the FI network covers ∼50% of the human proteome representing nearly 210,000 FIs. Of the 590 genes that were differentially expressed in response to DEX, 356 (60%) were projected onto the FI network. The average-shortest path calculation demonstrated that the interactions between the 365 genes were more highly connected with each other than by chance alone (p<0.001). A minimum spanning tree algorithm was used to add 128 “linker” genes from the FI network to create a single fully connected subnetwork [38]. A spectral partitioning clustering algorithm was then used to identify clusters of genes that were highly interconnected with each other [39]. The clustering algorithm identified 5 functionally related network modules (0 to 4), which consisted of 11 or more genes (Figure 3 and File S1). All network diagrams were visualized using Cytoscape [40]. Using functional enrichment analysis, the modules were annotated for BioCarta, KEGG, NCI-PID, Panther, and Reactome pathways with a false discovery rate (FDR)<0.05. Of interest were a number of pathways contained within the modules, including inflammatory response, interleukin and NFκB signaling (module 0; Figure S1 in File S1), chromosome maintenance and cell cycle (module 1; Figure S2 in File S1), integrin signaling and extracellular matrix organization (module 2; Figure S3 in File S1), ubiquitin mediated proteolysis and antigen processing/presentation (module 3; Figure S4 in File S1), and G protein coupled receptor signaling and downstream targets (module 4; Figure S5 in File S1). Using *NFKΒ1* and *RELA1* ‘linker’ genes of module 0, NFκΒ target genes and interacting proteins across four network modules were visualized in Cytoscape (Figure 4).

**Figure 5:**
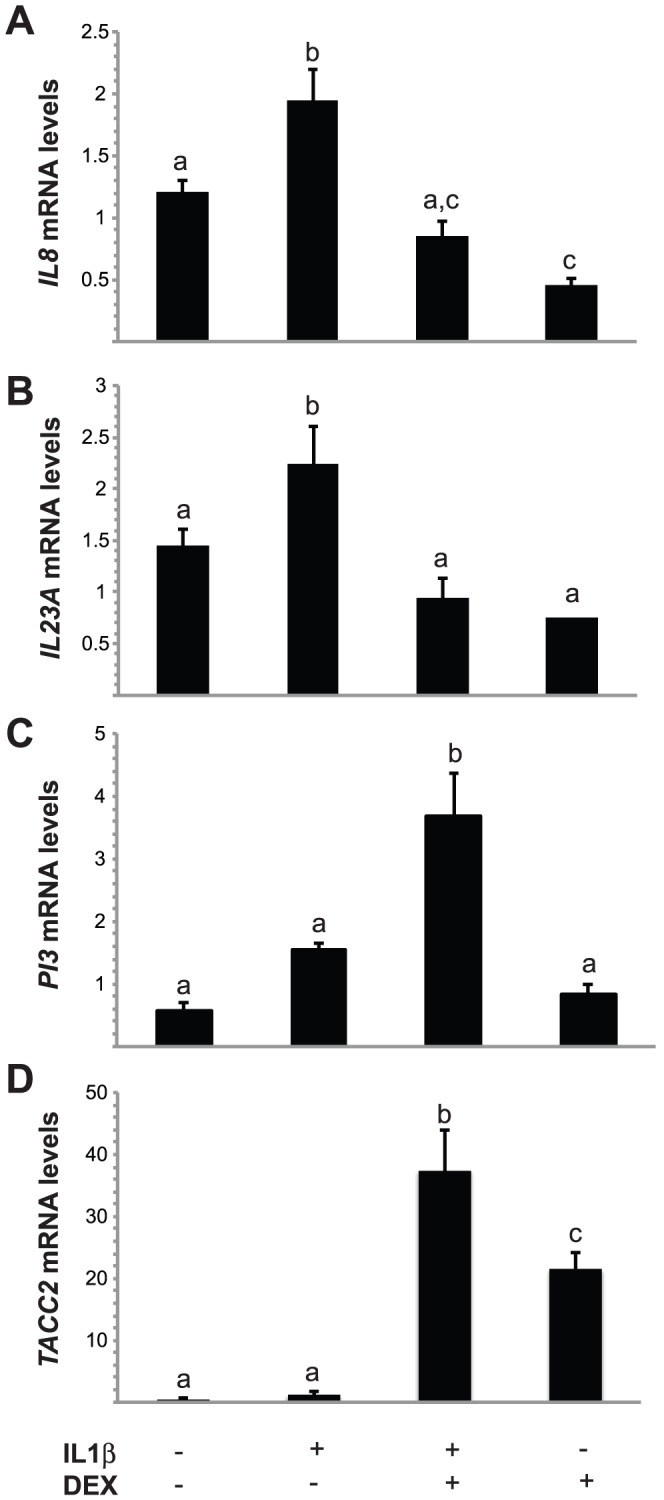
Transcript levels of *IL8*, *IL23A*, *PI3* and *TACC2* following IL1β and/or DEX treatment. OE-E6/E7 cells were treated with 10 nM DEX or vehicle 30 h prior to treatment with 50 ng/ml IL1β or vehicle and harvested 18 h later. Total RNA was extracted and RT-qPCR was performed for *IL8* (A), *IL23A* (B), *PI3* (C), *TACC2* (D) and were normalized to *TBP*. Bars represent the mean ± SEM (n = 3 biological replicates performed in triplicate). Bars with different letters are statistically different from one another as determined by ANOVA followed by a Student-Newman-Keuls post-hoc multiple comparison test (*p*<0.05).

Four genes were selected for validation as differentially expressed genes due to IL1β or DEX treatment based on their potential involvement in carcinogenesis. OE-E6/E7 cells were treated as described for the gene expression profiling study and levels of Interleukin 8 (*IL8*), Interleukin-23 alpha subunit p19 (*IL23A*), Peptidase Inhibitor 3 (*PI3*) and Transforming Acidic Coiled-Coil Containing protein 2 (*TACC2*) transcripts were measured by RT-qPCR. Microarray analysis indicated increased expression of NFκΒ target gene *IL23A* by IL1β that did not attain statistical significance with a FDR-corrected t-test (P = 0.051, Table S1); however, a statistically significant increase was detected by RT-qPCR (Figure 5B). A similar increase was also observed for the NFκΒ target gene *IL8* (Figure 5A). Consistent with an anti-inflammatory role, DEX pre-treatment inhibited the IL1β-induced expression of both genes. In agreement with our gene expression profiling data, DEX alone reduced expression of *IL8*. Also consistent with our microarray data, DEX treatment increased *TACC2* expression, which was further increased by IL1β (Figure 5D). *PI3* expression was increased by IL1β in our microarray analysis (Table 1) and the increase detected by RT-qPCR approached statistical significance (P = 0.096; Figure 5C). Consistent with our microarray data, increased *PI3* expression was detected with combined IL1β and DEX treatment.

**Figure 6:**
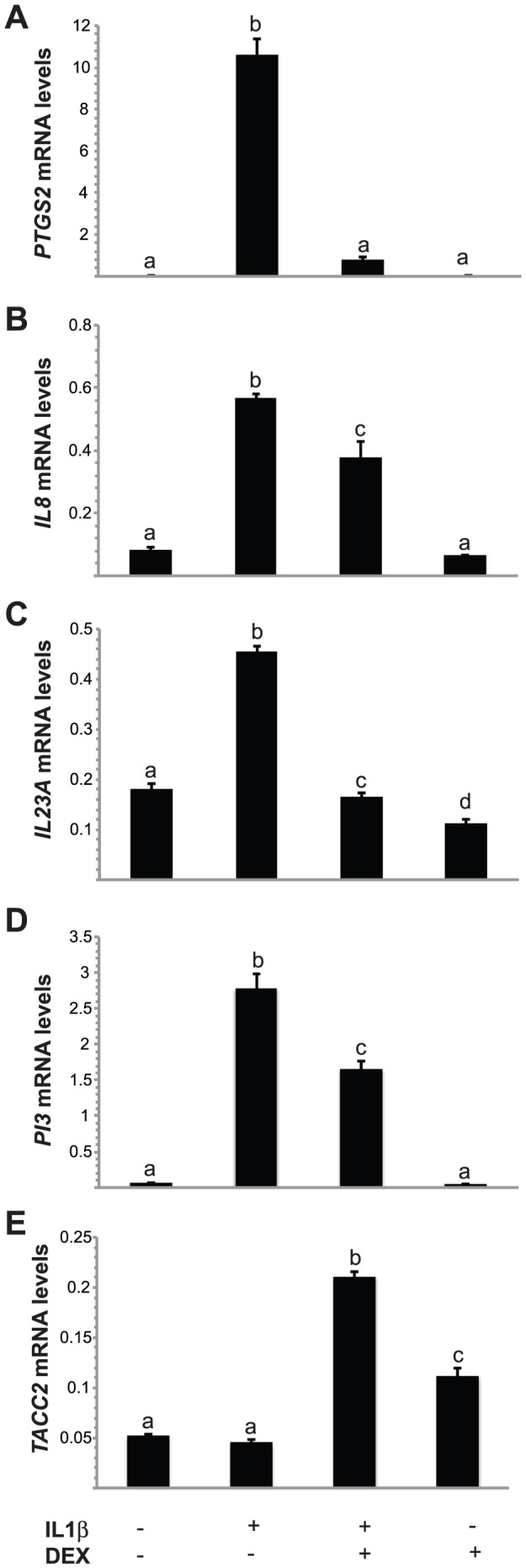
Transcript levels of *PTGS2*, *IL8*, *IL23A*, *PI3* and *TACC2* following IL1β and/or DEX treatment. Primary FTE cells were treated with 10/ml IL1β or vehicle and harvested 18 h later. Total RNA was extracted and RT-qPCR was performed for *PTGS2* (A), *IL8* (B), *IL23A* (C), *PI3* (D), *TACC2* (E) and were normalized to the geometric mean of 4 reference genes. Bars represent the mean ± SEM (n = 3). Bars with different letters are statistically different from one another as determined by ANOVA followed by a Student-Newman-Keuls post-hoc multiple comparison test (*p*<0.05).

To determine if similar effects are observed in non-immortalized FTE cells, a primary culture of human FTE cells derived from excess surgical material was treated with IL1β with or without DEX as described for OE-E6/E7 cells and RT-qPCR was performed for *PTGS2, IL8*, *IL23A*, *PI3* and *TACC2* transcripts. IL1β treatment increased the expression of *PTGS2, IL8*, *IL23A*, *PI3* transcripts and DEX treatment inhibited the IL1β-induced increase (Figure 6). DEX alone had no effect on *PTGS2, IL8*, or *PI3* transcript levels but decreased expression of *IL23A*. Similar to OE-E6/E7 cells, DEX treatment increased *TACC2* transcript levels, which were further elevated by IL1β co-treatment (Figure 6).

## Discussion

Inflammation plays an important role in the initiation and progression of several cancers [18], [41]. In healthy cycling women, the fallopian tube may be exposed to an inflammatory milieu at the time of ovulation and in the event of retrograde menstruation [42]. Ovulation is a highly-localized, acute inflammatory event that exposes the distal FTE to follicular fluid containing pro-inflammatory cytokines and mediators, including IL1 and TNF, as well as high concentrations of steroid hormones [43], [44]. Likewise, retrograde menstruation can expose the entire length of the fallopian tube to pro-inflammatory agents within the menses. Rapid resolution of inflammatory signaling is thus important to limit cellular damage to the epithelium. Approximately 80% of TICs within the FTE are found in the fimbria or distal portion of the tube [3], consistent with exposure to ovulation-associated inflammation. Molecular profiling of microdissected FTE from *BRCA* mutation carriers and control patients indicated luteal FTE from mutation carriers differed from that of control patients, whereas follicular FTE were highly similar between the two groups [45]. This difference in luteal phase FTE from *BRCA* mutation carriers predominantly reflected a pro-inflammatory gene signature consistent with increased NFκB signaling and diminished glucocorticoid receptor signaling [21], [46]. The FTE samples were not obtained immediately following ovulation and the response of tubule epithelial cells to inflammatory cytokine or glucocorticoid exposure has not been characterized.

NFκB, a principal intracellular mediator of pro-inflammatory cytokines, has been proposed as a mechanistic link between inflammation and cancer [47], [48] and its pro-inflammatory signaling targets are activated by both IL1 and TNF. Consistent with this, we found well-established direct transcriptional targets of NFκB signaling to be up-regulated by IL1β treatment in OE-E6/E7 cells.

Glucocorticoids exert pleiotropic effects critical to maintaining tissue homeostasis. As such, they play a key role in moderating and resolving inflammatory environments by inhibiting downstream signaling by inflammatory cytokines and by inducing cell-type specific apoptosis of monocytes, macrophages, and T-lymphocytes [49]. In addition to reversing the impact of IL1β on many inflammation-associated genes, our data indicate that glucocorticoid receptor signaling impacts the expression of multiple genes unaffected by IL1β. Activated glucocorticoid receptors heterodimerize with the NFκB subunit RelA, resulting in inhibition of RelA signaling [50], [51]. Additionally, the glucocorticoid receptor can affect the expression of anti-inflammatory genes through transactivation. Our pathway analysis indicated genes involved with inflammatory response, cell cycle, extracellular matrix organization, antigen processing, and G-protein coupled receptor signaling are affected by DEX treatment. Many of the genes in these pathways are NFκB target genes or encode NFκB interacting proteins. Several of these were not found to be affected by IL1β treatment, which could reflect differences in NFκΒ family members involved, the timing of the treatments, or regulation through independent pathways [52], [53]. Nonetheless, this finding is consistent with a more generalized anti-inflammatory effect of glucocorticoid receptor activaton that is not restricted to inhibition of IL1β-activated NFκΒ signaling.

The genes validated in this study – *PTGS2*, *IL8*, *IL23A*, *TACC2* and *PI3* – were selected based upon their differential response to treatment and their implication for cancer initiation. IL1 is a potent stimulator of *PTGS2* expression and prostaglandin production in ovarian tissue. Mice with targeted deletion of *PTGS2* fail to ovulate [54], indicating the importance of prostaglandin production in ovulation. Within the fallopian tube, prostaglandins may play a non-inflammatory role in muscular contraction and the resulting transport of the oocyte/embryo toward the uterus [55]. We found that treatment of OE-E6/E7 cells with peri-ovulatory follicular fluid collected from patients undergoing *in vitro* fertilization increased PTGS2 protein levels (data not shown). PTGS2 is a major inflammatory mediator up-regulated in several cancers, most notably colorectal carcinoma [56], [57]. Prostaglandins evoke further cytokine expression to promote the inflammatory response, which could contribute to carcinogenesis. Indeed, a protective effect of PTGS2 inhibition has been shown for certain cancers, such as colorectal cancer [56], [57]. Moreover, recent studies suggest aspirin, an inhibitor of both PTGS1 and 2, may exert a protective effect for invasive ovarian cancer [58], [59]. In the present study, we found PTGS2 expression was increased by IL1β, an effect that was blocked by glucocorticoid signaling. A stimulatory, rather than inhibitory effect of DEX on PTGS2 expression has been reported in a variety of cell types [22], [23], [24], [25]. While the mechanism underlying this paradoxical response are not fully understood, Wang et al [23] recently showed that DEX treatment of placental cytotrophoblast cells up-regulated expression of *MAP3K14/NIK*, a serine/threonine kinase that mediates activation of non-canonical NFκB signaling by processing NFκB2/p100 to active p52 [52]. In the present study, we did not see evidence of DEX altering the expression of *MAP3K14* or of NFκB subunits in OE-E6/E7 cells.

IL8 accentuates local pro-inflammatory signaling by attracting monocytes, neutrophils and T-cells and its expression is increased in the distal fallopian tube during the peri-ovulatory period [45], [60]. IL8 is elevated in the serum of ovarian cancer patients and enhances tumorigenicity of ovarian cancer cell mouse xenographs [61], [62]. Since *IL8* is a known downstream target of IL1 [26] and our profiling data indicated an inhibitory effect of DEX, we examined its expression by RT-qPCR. Our results are consistent with IL1β acting upon these cells and primary cultured FTE cells to induce *IL8*, and verified the inhibitory impact of DEX on this expression.

IL23 is a heterodimeric cytokine, consisting of a p19 (IL23A) and p40 (IL12β) subunit, produced by multiple cell types [63]. Although IL23 shares a subunit with IL12, it activates a distinct receptor and exerts effects that differ from those of IL12 [64]. In contrast to IL12, IL23 promotes pro-inflammatory responses and diminishes tumor infiltration of cytotoxic T cells as determined in a DMBA/TPA-induced skin papilloma mouse model [65]. More recently, Teng et al [66] showed IL23 promotes methylcholanthrene-induced fibrosarcoma in mice by antagonizing anti-tumor innate immune responses. This combination of stimulating inflammatory signaling while diminishing immune surveillance would facilitate early events in carcinogenesis. We found that IL1β stimulated expression of *IL23A* in both OE-E6/E7 and primary FTE cells and that this increase was blocked by DEX.

TACC2 is a member of a family of highly conserved proteins that associate with the centrosome-spindle apparatus and are thought to play a role in cell division [67]. In mammals, both TACC2 and 3 function as nuclear receptor coactivators [60], [67]. TACC2 associates with retinoic acid receptor RXRβ and with histone acetytransferases and the Switch/sucrose nonfermentable (swi/snf) complex involved with chromatin remodeling and transcription [67], [68], [69]. While both tumor promoting and suppressive activities of TACC2 have been reported in various cancers [70], [71], [72], Lauffart et al [73] have shown that TACC2 and 3 interact with the BARD1/BRCA1 complex and may play a role in DNA double-strand break repair. Our data indicate *TACC2* expression is increased by DEX in both OE-E6/E7 cells and primary FTE cells, which is consistent with a previous report showing increased expression of TACC2 by DEX in ovarian cancer cells [74]. The functional impact of this increase remains to be determined.

*PI3* encodes a serine protease inhibitor that functions as an anti-inflammatory mediator. Ghosh et al [75] reported that epithelial cells within the female reproductive tract, including the fallopian tube, express PI3 with higher levels observed during the luteal phase. Although PI3 has been shown to reduce neutrophil and macrophage accumulation and increase G-CSF levels, consistent with a role in innate immunity, a role in adaptive immunity has also been suggested [76]. Consistent with our data showing increased PI3 expression by IL1β, other studies have shown increased expression of PI3 in IL1β-treated endocervical cells and in OE-E6/E7 cells exposed to *Chlamydia trachomatis* [77], [78], likely through NFκB signaling [79]. Since NFκB signaling is in turn inhibited by PI3 [80] this increase in PI3 may be directed at containing the pro-inflammatory response. Our finding that DEX treatment further increased PI3 expression in the presence of IL1β in OE-E6/E7 cells was surprising, given the inhibitory impact of glucocorticoid activation on NFκB signaling. However, an inhibitory effect of DEX on IL1β-induced *PI3* transcript levels was observed in primary FTE cells.

In summary, we demonstrate that IL1β, a cytokine associated with ovulation, induces a pro-inflammatory gene expression signature in an immortalized human fallopian tube epithelial cell line. Activation of glucocorticoid receptor by a synthetic agonist reversed the expression of several IL1β-induced inflammatory genes and altered the expression of additional inflammation-associated genes. These findings were confirmed for key representative genes in a primary culture of FTE cells. Thus, this study provides support for the hypothesis that pro-inflammatory signaling is induced in FTE cells by inflammatory mediators, such as those present in follicular fluid at the time of ovulation, and that this signaling is opposed by glucocorticoids. Dysregulation of glucocorticoid receptor signaling could therefore contribute to increased risk for HGSOC.

## Supporting Information

Table S1 **Annotation of Genes in Venn diagram shown in** **Figure 2** **.**
(XLSX)

File S1 **Supporting Figures. Figure S1.** Network Module 0, representing ‘Inflammatory response, Interleukin signaling and NFκB signaling’ shown in Figure 3. **Figure S2.** Network Module 1, representing ‘Chromosomal maintenance and Cell cycle’ shown in Figure 3. **Figure S3.** Network Module 2, representing ‘Integrin signaling and Extracellular matrix organization’ shown in Figure 3. **Figure S4.** Network Module 3, representing ‘Ubiquitin mediated proteolysis and Antigen processing and presentation’ shown in Figure 3. **Figure S5.** Network Module 4, representing ‘G protein coupled receptor signaling and downstream targets' shown in Figure 3.
(DOC)
