# Supplementary material for: Glucocorticoid-Induced Reversal of Interleukin-1β-Stimulated Inflammatory Gene Expression in Human Oviductal Cells
Source: PLoS One. 2014 May 21;9(5):e97997. doi: 10.1371/journal.pone.0097997 (PMC4029821; doi:10.1371/journal.pone.0097997)
Supplement: File S1 — Supporting Figures. Figure S1. Network Module 0, representing ‘Inflammatory response, Interleukin signaling and NFκB signaling’ shown in Figure 3. Figure S2. Network Module 1, representing ‘Chromosomal maintenance and Cell cycle’ shown in Figure 3. Figure S3. Network Module 2, representing ‘Integrin signaling and Extracellular matrix organization’ shown in Figure 3. Figure S4. Network Module 3, representing ‘Ubiquitin mediated proteolysis and Antigen processing and presentation’ shown in Figure 3. Figure S5. Network Module 4, representing ‘G protein coupled receptor signaling and downstream targets' shown in Figure 3. (DOC) [file pone.0097997.s002.doc]

**SuppORTING FIGUREs FILE S1**

**Figure S1**. **Network Module 0, representing ‘Inflammatory response, Interleukin signaling and NFkB signaling’ shown in Figure 3.** Genes up-regulated in human FTE OE-E6/E7 cells treated with DEX are depicted by red outer circles, whereas genes down-regulated in human FTE OE-E6/E7 cells treated with DEX are depicted by green outer circles. Linker genes are depicted as diamond shaped nodes. Direct activating or inhibitory interactions are indicated with the symbols → and -|, respectively. Indirect interactions involving additional proteins are depicted with dashed lines.

**Figure S2. Network Module 1, representing ‘Chromosomal maintenance and Cell cycle’ shown in Figure 3.**  Genes up-regulated in human FTE OE-E6/E7 cells treated with DEX are depicted by red outer circles, whereas genes down-regulated in human FTE OE-E6/E7 cells treated with DEX are depicted by green outer circles. Linker genes are depicted as diamond shaped nodes. Direct activating or inhibitory interactions are indicated with the symbols → and -|, respectively. Indirect interactions involving additional proteins are depicted with dashed lines.

**Figure S3. Network Module 2, representing ‘Integrin signaling and Extracellular matrix organization’ shown in Figure 3.** Genes up-regulated in human FTE OE-E6/E7 cells treated with DEX are depicted by red outer circles, whereas genes down-regulated in human FTE OE-E6/E7 cells treated with DEX are depicted by green outer circles. Linker genes are depicted as diamond shaped nodes. Direct activating or inhibitory interactions are indicated with the symbols → and -|, respectively. Indirect interactions involving additional proteins are depicted with dashed lines.

**Figure S4. Network Module 3, representing ‘Ubiquitin mediated proteolysis and Antigen processing and presentation’ shown in Figure 3.**  Genes up-regulated in human FTE OE-E6/E7 cells treated with DEX are depicted by red outer circles, whereas genes down-regulated in human FTE OE-E6/E7 cells treated with DEX are depicted by green outer circles. Linker genes are depicted as diamond shaped nodes. Direct activating or inhibitory interactions are indicated with the symbols → and -|, respectively. Indirect interactions involving additional proteins are depicted with dashed lines.

**Figure S5. Network Module 4, representing ‘G protein coupled receptor signaling and downstream targets’ shown in Figure 3.** Genes up-regulated in human FTE OE-E6/E7 cells treated with DEX are depicted by red outer circles, whereas genes down-regulated in human FTE OE-E6/E7 cells treated with DEX are depicted by green outer circles. Linker genes are depicted as diamond shaped nodes. Direct activating or inhibitory interactions are indicated with the symbols → and -|, respectively. Indirect interactions involving additional proteins are depicted with dashed lines.
